# Molecular Epidemiology of Rhinovirus/Enterovirus and Their Role on Cause Severe and Prolonged Infection in Hospitalized Patients

**DOI:** 10.3390/microorganisms10040755

**Published:** 2022-03-31

**Authors:** Federica A. M. Giardina, Antonio Piralla, Guglielmo Ferrari, Federica Zavaglio, Irene Cassaniti, Fausto Baldanti

**Affiliations:** 1 Microbiology and Virology Department, Fondazione IRCCS Policlinico San Matteo, 27100 Pavia, Italy; federica.giardina01@universitadipavia.it (F.A.M.G.); guglielmo.ferrari01@universitadipavia.it (G.F.); fede.zavaglio90@gmail.com (F.Z.); i.cassaniti@smatteo.pv.it (I.C.); fausto.baldanti@unipv.it (F.B.); 2 Department of Clinical-Surgical, Diagnostic and Pediatric Sciences, Università degli Studi di Pavia, 27100 Pavia, Italy

**Keywords:** respiratory infection, rhinovirus, enterovirus, prolonged infection

## Abstract

Rhinovirus is one of the most common respiratory viruses, causing both upper and lower respiratory tract infections. It affects mainly children and could cause prolonged infections, especially in immunocompromised patients. Here we report our data on a 15-month surveillance of Rhinovirus seasonality and circulation in Lombardy Region, Italy. All rhinovirus/enterovirus-positive samples were amplified with RT-PCR for the VP4-VP2 region to assign the correct genotype. The median age of RV/EV-positive patients is 9 years, with a range of 0–96. RV-A and RV-C were detected in the majority of cases, while RV-B accounted for less than 10% of cases. An enterovirus species was detected in 6.45% of the cases. A total of 7% of the patients included in this study had a prolonged infection with a median duration of 62 days. All these patients were immunocompromised and most of them were pediatric with an RV-A infection. Two outbreaks were identified, mainly in the neonatal intensive care unit (NICU) and Oncohematology Department, caused by RV A89 and C43, respectively. Nearly 4.5% of the patients were admitted to the ICU requiring mechanical ventilation; all of which had preexisting comorbidities.

## 1. Introduction

Human rhinoviruses (RVs) are small viruses belonging to the Enterovirus genus within the Picornaviridae family. At least more than 100 genotypes have been identified and recently a new species of RV, named RV-C, including more than 50 genotypes, was firstly discovered in 2006 [1]. RV infections are quite common and their circulation seems to be distributed into two yearly peaks; in early fall and spring [2]. In particular, RV-A and RV-C represent the species most frequently detected, while RV-B is less frequent in comparison.

Although RVs are considered the etiologic agents of the “common cold”, RVs have been recently associated with a severe acute respiratory infection (SARI) in children, older people, and immunosuppressed subjects [3,4,5]. Clinical manifestations of RV-associated SARI are croup, bronchiolitis or community-acquired pneumonia (CAP) which often requires hospitalization and mechanical ventilation [6,7,8]. Among RV species, RV-C seems to be more frequently associated with severe infections [9] including asthma exacerbations in children and life-threatening conditions in infants [10,11]. Other studies suggest that RV-C is more likely to cause lower respiratory tract infections than other types of RVs in the pediatric population rather than in the adult population [12]. Furthermore, RVs are also implicated in nosocomial outbreaks, as observed in neonatal intensive care units [13,14]. In addition to RVs, enteroviruses (EVs) belonging to the same Picornaviridae family have been observed as emerging pathogens causing a wide range of clinical syndromes, ranging from mild to more severe clinical outcomes [15,16].

RV and EV shedding usually lasts less than 2 weeks in immunocompetent subjects [17], while prolonged RV infections have been mainly observed in those who are immunocompromised, such as patients undergoing chemotherapy or in a post-transplant phase [18].

This study aimed to investigate clinical and virological features of RV/EV infections providing the increasingly recognized role of these viruses as important disease-causing agents in order to describe their impact on short- and long-term morbidity.

## 2. Materials and Methods

### 2.1. Study Population

This study was conducted in a cohort of patients with a respiratory syndrome, both hospitalized and outpatients, all referring to the Fondazione I.R.C.C.S. Policlinico San Matteo hospital in Pavia, Italy. All respiratory samples (nasal swabs and bronchoalveolar lavages) were collected between 1 September 2017, and 31 December 2018, then analyzed for the presence of respiratory viruses. Patients with rhinitis, pharyngitis, and laryngitis were considered as affected by an upper respiratory tract infection (URTI), whereas patients with bronchitis, bronchiolitis, and pneumonia (characterized by cough, wheezing, and/or dyspnea) were classified as affected by a lower respiratory tract infection (LRTI). In addition to the suggestive clinical picture, all cases of pneumonia were radiologically confirmed. The term “episode” indicated a single respiratory syndrome, whose duration was defined by the presence of respiratory symptoms. Respiratory syndromes occurring in the same patient at least 3 weeks following the disappearance of respiratory symptoms of a previous episode were defined as a “separate episode” and were analyzed independently from the previous one. “Multiple picornavirus detection” stated the presence of different RV/EV strains or species during the same episode. Episodes were defined as “prolonged” if the same RV/EV type was detected in specimens collected at least 30 days apart. The study was conducted in accordance with the Declaration of Helsinki, and the protocol on respiratory virus epidemiology was approved by the Ethics Committee of our hospital (P-20180022616).

### 2.2. Molecular Analysis

Viral RNA was extracted on the QiaSymphony platform using a Virus Pathogens DSP Midi Kit (Qiagen, Heidelberg, Germany). Clinical specimens were tested for respiratory viruses using a panel which included RV/EV, human influenza type A and B (FluA and FluB), human coronaviruses (hCoVs), human metapneumovirus (hMPV), human respiratory syncytial virus (hRSV), human parainfluenza virus (hPiV) type 1–4 and human adenoviruses (hAdV) [19]. Real-time RT-PCR reactions were performed on Rotor-Gene Q with a Quantifast^®^ Pathogen PCR+IC Kit (Qiagen, Heidelberg, Germany), according to the manufacturer’s instructions. RV/EV-positive samples were amplified in a nested PCR targeting the VP4-VP2 region of the viral genome, according to Wisdom et al. [20], with a modified protocol. In detail, the first amplification was performed using the AgPath-ID One-Step RT-PCR kit (Ambion, Austin, TX, USA), according to the manufacturer’s instructions. Primers used in the first amplification were OS458 (5′CCGGCCCCTGAATGYGGCTAA3′) and OAS1125 (5′ACATRTTYTSNCCAAANAYDCCCAT3′). Thermal profile was as follows: retrotranscription was conducted at 50 °C for 30 min and initial PCR activation at 95 °C for 10 min, then 50 cycles at 95 °C for the 30 s, 58 °C for 30 s and 72 °C for 1 min. The final step was at 72 °C for 5 min. Nested amplification was performed using AmpliTaqGold^®^ with GeneAmp^®^ (Life Technologies, Livingston, NJ, USA) according to the manufacturer’s instructions with primers IS547 (5′ACCRACTACTTTGGGTGTCCGTG3′) and IAS1087 (5′TCWGGHARYTTCCAMCACCANCC3′). The thermal profile was: 95 °C for 10 min followed by 40 cycles at 95 °C for 30 s, 58 °C for 30 s, 72 °C for 1 min. The final step of the reaction was at 72 °C for 5 min.

An alternative protocol targeting the EV’s VP1 protein was used whenever the direct typing PCR resulted negative as described by Nix and colleagues [21]. The sequencing reaction was performed using internal primers on an ABI Prism Genetic Analyzer and sequences obtained were analyzed on Sequencer software.

### 2.3. Phylogenetic Analysis

Nucleotide sequences were aligned using the ClustalW method (we applied the ClustalW method for the nucleotide sequences’ alignment) and a phylogenetic tree was constructed using the neighbor-joining method and the kimura-2-parameter to simultaneously estimate the distance among the sequences with MEGA software (version 5.05) [22]. Bootstrap values included 1000 replicates. RV/EV type assignment was defined by the nearest reference strains observed in the phylogenetic tree (The RV/EV type was assigned taking into account the nearest reference strain/s observed in the phylogenetic tree).

### 2.4. Statistical Analysis

Comparisons of the continuous unpaired variables were performed with the Mann–Whitney test. Additionally, we carried out descriptive statistics and statistical comparisons using the Graph Pad Prism software (version 8.3.0). 

## 3. Results

### 3.1. Samples

**Figure 1:**
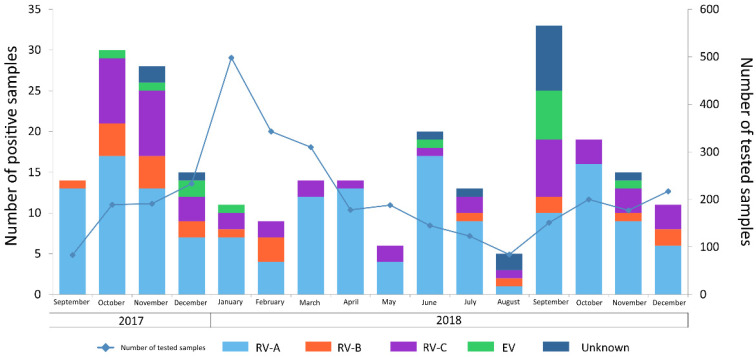
Monthly distribution of cases included in this study. The blue line represents the total number of respiratory specimens collected and tested during the study period. The bars correspond to the RV/EV positive cases with RV-A reported in light blue, RV-B in orange, RV-C in violet and EV cases in green. Blue bars represent those cases for which typing was not possible due to a very low viral load.

A total of 3310 respiratory specimens were collected from hospitalized patients and outpatients and then analyzed during the study period. As shown by the blue line in Figure 1, the highest number of specimens was collected in December 2017 and January 2018, at the beginning of the influenza viruses’ circulation. Instead, the lowest number of specimens was collected and tested during the summer period in 2018. Of the 3310 specimens collected, 257 (7.6%) referred to 201 patients (8.9% of the total), were positive for RV/EV (177 nasal swabs, 45 nasopharyngeal swabs, and 35 bronchoalveolar lavages). A total of 127/201 (63.2%) patients were admitted to different departments of our hospital, including Infectious Disease Dept., Pediatrics Dept., Oncohematology Dept., ICU or NICU Dept. A total of 66/201 (32.8%) were instead outpatients. Regarding the remaining 8 (4%) RV/EV positive cases, specimens were sent to our hospital from other health institutes in the Lombardy Region. The median age of RV/EV-positive patients was 9 years old (range 10 days–96 years), including 117 males (58.2%) and 84 females (41.8%). Of the total, 188/201 (93.5%) patients had one single RV/EV episode, 11 (5.5%) had 2 different RVRV/EV episodes, and only 2 (1%) patients had 3 RV/EV episodes. Among 216 episodes of RV infection, 184/216 (85.2%) were URTI and 32/216 (14.8%) were LRTI.

Table 1 shows the demographic and virological features of the patients included in this study. In the 3.053 RV/EV negative specimens, hRSV was detected in 274 samples (8.3%), FluB in 126 (3.8%), FluA in 106 (3.2%), hPiV type 1/3 and hAdV in 65 samples each (2.0%), hMPV in 37 (1.1%) and hCoV type OC43/HKU1 in 24 (0.7%). hPiV type 2/4 and hCoV 229E/NL63 represented less than 0.5% of the total number of cases and were detected in 13, 4 and 5 specimens, respectively. Finally, 2334 (73.2%) samples resulted negative for the respiratory viruses included in the panel used.

### 3.2. The Peak of Viral Load and Prolonged Infection

The peak of the RV/EV load was between 10^3^ and 10^5^ copies/mL in 51.9% of the episodes (112/216), >10^5^ copies/mL in 53/216 (24.5%) and lower than 10^3^ copies/mL in the remaining 51/216 (23.6%) episodes.

Overall, the median duration of RV/EV episodes was 15 days (range 4–316 days). A total of 11 (5.5%) patients had a prolonged RV/EV infection (>30 days) with a median duration of 75 days (range 30–316 days). All of these patients were immunocompromised due to their age (<30 days old), ongoing chemotherapy or post-transplant immunosuppressive therapy.

### 3.3. Typing and Coinfections

**Figure 2:**
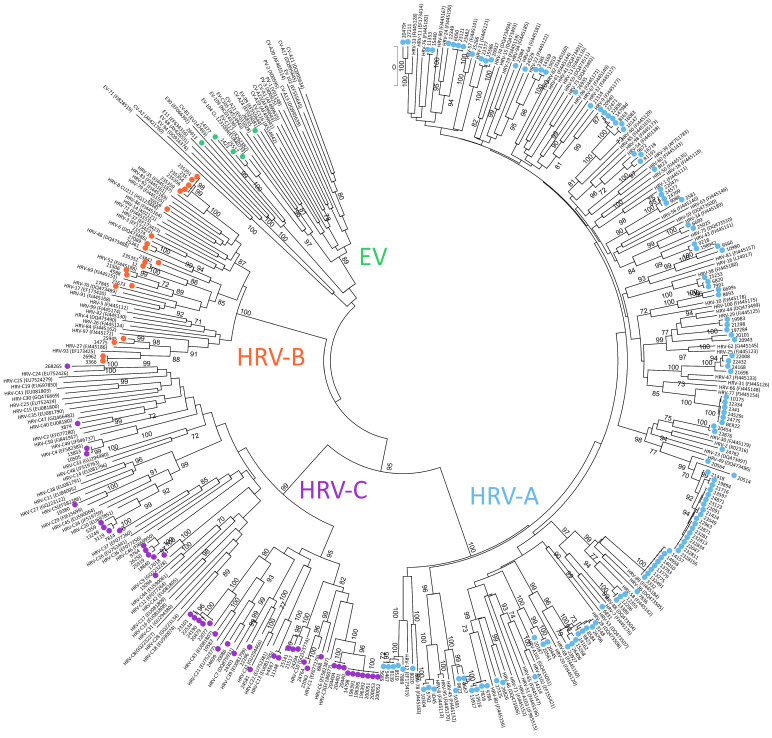
Shows the phylogenetic tree of the RV/EV cases in this study, based on the VP4-VP2 sequences obtained (*n* = 196). RV-A strains are reported with light blue circles, RV-B with orange circles, RV-C with violet circles and EV cases with green circles. EV-D68 sequences are not reported here due to the different sequences (partial VP1) analyzed.

Out of 216 RV/EV episodes, 127 (58.3%) were caused by RV-A, 44 (19.9%) by RV-C and 20 (9.8%) by RV-B, and the remaining 12 (6.0%) episodes were caused by EV (Figure 2).

In regard to the RV-A positive cases, 40 different genotypes were detected, 8 genotypes for RV-B and 18 for RV-C. For each RV species, the most detected genotypes were A49, B35 and C3, respectively. Concerning the EV-associated episodes, an EV-D68 was detected in seven of them, an EV-C104 was detected in two, and one single identification was obtained for EV-C117, CV-A21, and CV-B4. Among the 216 cases of infection, one subject was simultaneously infected by RV-A and EV-D68. In 14 RV/EV episodes typing could not be performed due to a very low viral load.

The median age of RV-A positive pediatric patients was 22 months, 29 months for RV-B, and 7 months for RV-C. Mann–Whitney tests showed that the median age between RV-A/B and RV-A/C positive pediatric patients was significantly different (*p* < 0.05), and that RV-A strains were observed in older people (16–65 and >65 years) as compared to RV-B and RV-C (Table 1). In 175/216 episodes (81.0%), RV/EV was the only virus detected, however, in 41/216 (19.0%) RV/EV was detected with at least one other respiratory virus. In detail, hRSV was detected in 24/41 (58.5%) coinfections, hAdV in 4/41 (9.8%), hPiV3 in 4/41 (9.8%), hMPV in 2/41 (4.9%), hPiV4 in 2/41 (4.97%) and finally, RV/EV was detected with two other viruses (hPiV and hCoV) in only one sample. Given the retrospective nature of this study, bacterial and fungal coinfections were not investigated.

### 3.4. Hospital Outbreaks

During the study period, at least two nosocomial outbreaks were observed in our hospital. The first outbreak occurred in October 2017 in a neonatal intensive care unit (NICU), with four patients infected by RV-C43 in a 30-day period. The other outbreak occurred in the same NICU in June 2018 where four patients were infected by RV-A89 during the same period (12 days).

### 3.5. EV Episodes Associated with Severe Infection

A total of 13 (4.5% of all the positive patients) RV/EV-positive patients were admitted to the ICU with a severe respiratory acute infection (SARI). The median age of the ICU patients was 54 years (range 5–66 years) including five patients with age less than 11 years. In all the cases, mechanical ventilation was needed. Eleven out of 13 patients (84.6%) had preexisting comorbidities including chronic respiratory diseases, and hematologic malignancies such as acute lymphoblastic leukemia, lymphoma and myelodysplastic syndrome. RV/EV was detected in bronchoalveolar lavage in 8/13 (61.5%) patients, nasal swab in 3/13 (23%) and in both samples in two patients. In six BALs, RV was detected with a viral load between 10^4^–10^5^ copies/mL, while in five BALs, the viral load was lower than 10^4^ copies/mL. In one BAL, the RV/EV viral load was higher than 10^6^ copies/mL. In seven patients, RV/EV was the only respiratory pathogen detected including four RV-A two RV-B, and one RV-C. On the contrary, in six patients, RV/EV (including three RV-A and three RV-B) was simultaneously detected with CMV (three patients), hAdV (one patient), hPiV3 (one patient), and hPiV4 (one patient). Bacterial/fungal coinfections were detected in two patients, one with *S. pneumoniae* (also positive for CMV) and one with both *S. pneumoniae* and *P. aeruginosa.*

## 4. Discussion

Influenza viruses and RSV are well-known respiratory pathogens, while RV/EV are increasingly recognized pathogens also responsible for SARI. In our study, 8.9% of all the patients referring to our hospital with acute respiratory syndromes had an RV/EV infection. Nearly 60% of them were pediatric (age < 16 years) and this finding is in agreement with recent studies investigating the epidemiology of respiratory infections. Those studies show that most RV/EV-positive patients were children under 10 years of age or less [23] and a difference in the median age in children infected by different RV genotypes has been observed [24,25,26]. Most of the episodes of RV infections observed in this study were caused by RV genotypes A and C, while RV-B accounts for about 10% of the total episodes. As previously described in other studies, comparable distribution of RV species was observed worldwide, with a high incidence of RV-A and RV-C often present in equal or similar proportions [27,28]. The most frequently detected viruses in coinfections were RSV and AdV, while influenza viruses were never detected in coinfections with RV/EV. This finding is in agreement with many other studies investigating respiratory viruses’ epidemiology, in which the viruses most frequently detected in coinfections with RV/EV were AdV and RSV together with human bocavirus [26,29]. In our study, the rate of coinfection with at least one other virus is 23.4%. Other studies reported frequencies of viral coinfection ranging from 9 [30] to 47% [31].

During our study period, two intra-hospital outbreaks were observed in October 2017 and June 2018. Molecular epidemiology of RV/EV has allowed us to identify several outbreaks in neonatal settings, including patients requiring mechanical ventilation [14,32,33]. Since RVs spread via aerosolization or direct contact with an infected person, intra-hospital outbreaks could be caused by contaminated surfaces or staff members during viral shedding following symptoms resolution, as has been assumed by Reese and colleagues [13].

In immunosuppressed patients, RV/EV shedding has been observed for a prolonged period, and this occurrence seems to be correlated with an early phase post-transplant [17,34]. This prolonged infection has been sustained by active viral excretion that can last several months [35].

In our study, twelve cases of prolonged RV/EV infection were observed. The great majority of them were observed in patients undergoing chemotherapy or post-transplant therapy, or in newborns. In most cases, the clinical picture associated with this prolonged infection was mild, but few severe cases were also observed. A similar scenario was also described in patients with prolonged RV infection after lung transplant; most of whom were asymptomatic [36].

Although RV has been considered the causative agent of the common cold, in our study 4.5% of RV/EV-positive patients had pneumonia and were admitted to the ICU. This finding has been recently investigated in the contest of other respiratory viruses. In 2015, Jain and colleagues reported that RV was the virus most frequently detected among ICU adult patients in the U.S., while in pediatric patients, RV was the second after hRSV, even if they were detected with a similar percentage [37,38]. Similar results were reported in Europe [39,40] as well as in Asia [41]. All these studies underline the need to consider RV/EV as the causative agent of severe respiratory infections. Sometimes severe RV/EV infections are also observed in patients with pre-existing comorbidities [17,38,42].

Nearly 60% of severe RV/EV infections were diagnosed based on LRT samples: this finding highlights the importance of adequate sampling collection. In fact, when both URT and LRT samples of the same patient were analyzed for the presence of RV/EV, it was detected only in LRT samples [19,43,44].

This study has several limitations. It is retrospective, and little data about patients’ clinical conditions are available. Information regarding bacterial and fungal coinfection was available only for severe cases and they were not investigated in the general population.

## 5. Conclusions

RVs/EVs circulate throughout the year, causing upper respiratory tract infections in immunocompetent subjects. However, they could cause prolonged and severe infections requiring ICU admission in high-risk patients such as the older or immunocompromised populations. For this reason, RV/EV infection should be systematically monitored. The clinical impact of RV/EV infections is not limited only to the common cold, and these viruses should be considered as highly significant respiratory pathogens.

## Figures and Tables

**Table 1:** Demographic and virological features of all RV/EV episodes with successful typing.

Categories		RV Species				*p*-Value ^a^
		RV-A (127)	RV-B (20)	RV-C (44)	EV (12)	
Gender	Male	77 (60.6%)	10 (50.0%)	28 (63.6%)	5 (41.7%)	0.57
	Female	50 (39.4%)	10 (50.0%)	16 (36.4%)	7 (58.3%)	
Age	<1 year	26 (20.5%)	4 (20.0%)	17 (38.6%)	3 (25.0%)	0.02
	1–5 years	24 (18.9%)	7 (35.0%)	13 (29.5%)	2 (16.7%)	
	5–15 years	20 (15.7%)	4 (20.0%)	0	0	
	16–65 years	43 (33.9%)	4 (20.0%)	10 (22.7%)	5 (41.7%)	
	>65 years	14 (11.0%)	1 (5.0%)	4 (9.1%)	2 (16.7%)	
Hospitalization	ICU Dept.	5 (3.9%)	3 (15.0%)	2 (4.5%)	0	0.10
	NICU Dept.	19 (15.0%)	2 (10.0%)	13 (29.5%)	2 (16.7%)	
	Infectious Diseases Dept.	4 (3.1%)	0	2 (2.5%)	2 (16.7%)	
	Other Depts.	96 (75.6%)	14 (70%)	27 (61.4%)	6 (50%)	
	Unknown	3 (2.4%)	1 (5.0%)	0	2 (16.7%)	
Immuno status	Immunocompromised	57 (44.9%)	6 (30.0%)	20 (45.5%)	5 (41.7%)	0.67
	Immunocompetent	50 (39.4%)	11 (55%)	16 (36.4%)	3 (25%)	
	Unknown	20 (15.7%)	3 (15%)	8 (18.2%)	4 (33.3%)	
Viral Load	<10^3^ copies/ml	25 (19.7%)	5 (25%)	12 (27.3%)	1 (8.3%)	0.46
	10^3^–10^5^ copies/mL	68 (53.5%)	13 (65.0%)	22 (50.0%)	10 (83.3%)	
	>10^5^ copies/mL	34 (26.8%)	2 (10.0%)	10 (22.7%)	1 (8.3%)	
Coinfections	No coinfections	106 (83.5%)	12 (60%)	36 (81.8%)	10 (83.3%)	
	Coinfections	21 (16.5%)	8 (40.0%)	8 (18.2%)	2 (16.7%)	
	hADV	0	2 (10%)	2 (4.5%)	0	0.17
	hCMV	6 (4.7%)	1 (5%)	1 (2.3%)	1 (8.3%)	
	hCOVs	1 (0.8%)	0	0	0	
	hMPV	1 (0.8%)	0	1 (2.3%)	0	
	hPIVs	2 (1.6%)	3 (15.0%)	0	0	
	hRSV	11 (8.7%)	2 (10.0%)	4 (9.1%)	1 (8.3%)	
Genotypes	N. of detected genotypes	40	9	20	5	NA
	Unknown	7	6	4	1	NA
	Most detected genotype	A49	B35	C3	D68	NA

^a^ *p*-value was calculated for comparison between RV-A, RV-B, and RV-C; NA, not applicable.

## Data Availability

Not applicable.
